# Amyloid formation of growth hormone in presence of zinc: Relevance to its storage in secretory granules

**DOI:** 10.1038/srep23370

**Published:** 2016-03-23

**Authors:** Reeba S. Jacob, Subhadeep Das, Saikat Ghosh, Arunagiri Anoop, Narendra Nath Jha, Tuhin Khan, Praful Singru, Ashutosh Kumar, Samir K. Maji

**Affiliations:** 1 Department of Biosciences and Bioengineering, IIT Bombay Mumbai, India 400 076; 2 IITB Monash Research Academy, IIT Bombay, Powai, Mumbai 400076, India; 3 Department of Chemistry, IIT Bombay Mumbai, India 400076; 4 School of Biological Sciences, National Institute of Science Education and Research, Bhubaneswar, India

## Abstract

Amyloids are cross-β-sheet fibrillar aggregates, associated with various human diseases and native functions such as protein/peptide hormone storage inside secretory granules of neuroendocrine cells. In the current study, using amyloid detecting agents, we show that growth hormone (GH) could be stored as amyloid in the pituitary of rat. Moreover, to demonstrate the formation of GH amyloid *in vitro*, we studied various conditions (solvents, glycosaminoglycans, salts and metal ions) and found that in presence of zinc metal ions (Zn(II)), GH formed short curvy fibrils. The amyloidogenic nature of these fibrils was examined by Thioflavin T binding, Congo Red binding, transmission electron microscopy and X-ray diffraction. Our biophysical studies also suggest that Zn(II) initiates the early oligomerization of GH that eventually facilitates the fibrillation process. Furthermore, using immunofluorescence study of pituitary tissue, we show that GH in pituitary significantly co-localizes with Zn(II), suggesting the probable role of zinc in GH aggregation within secretory granules. We also found that GH amyloid formed *in vitro* is capable of releasing monomers. The study will help to understand the possible mechanism of GH storage, its regulation and monomer release from the somatotrophs of anterior pituitary.

Amyloid fibril formation is associated with many human diseases such as Alzheimer’s and Parkinson’s^1^. During amyloid formation, both natively structured and unstructured proteins self-associate to form soluble oligomers and eventually convert to β-sheet rich fibrils^2^. Protein/peptides, irrespective of their disease association can form amyloids suggesting that amyloid formation may be a generic property of polypeptide chains^1^. Moreover, recent studies have suggested that many organisms (from yeast to mammals) utilize regulated amyloid formation to carry out their native functions^1^,^3^. For example, curli fibrils of *E. coli* are essential for surface attachment and host infectivity of the bacteria^4^. Pmel 17 amyloid is required for the regulated melanin polymerization for melanosome biogenesis in mammals^5^,^6^. Protein/peptide associations into highly organized intermolecular aggregates are implicated in the pathway of regulated secretion during secretory granule biogenesis^7^,^8^. Previous reports suggest that these aggregates are mostly stable against higher temperature, enzymatic degradation, mild detergent and large pH range^9^,^10^ and are suggested to possess crystalline structure^11^,^12^,^13^. However, it was recently suggested that these protein/peptide aggregates in secretory granules are amyloidogenic in nature^14^. Therefore, studying the aggregation of protein/peptide hormones destined for regulated secretion is important for understanding the mechanism of hormone storage.

Human growth hormone (GH) is a 191 residue long helical protein, which is essential for various functions including growth and metabolism of mammals^15^,^16^. This hormone is synthesized, stored, and secreted by the somatotroph cells in the anterior pituitary gland^17^,^18^. The storage of GH in secretory granules involves only minimum processing; hence the biogenesis of GH secretory granules is considered as a model system to study secretory granule formation^19^. GH release is highly regulated by two other hormones; growth hormone releasing hormone (GHRH)^20^ and somatostatin^21^. The balance between these two hormones maintains the level of GH in blood. The dysregulation of GH storage and secretion causes many human diseases. For example, hypersecretion of GH by somatotroph cells, present in pituitary tumors, causes acromegaly in adults^22^,^23^. Further, GH deficiency causes growth failure and short stature in children and various other problems including decreased energy and quality of life in adults^24^. It was previously reported that GH mutant (R183H) is able to form secretory granules in cells and has normal biological functions^25^,^26^. However, the secretory granules of mutant hormone are not able to release monomeric hormone from secretory granules, which may cause GH deficiency syndromes^25^,^26^,^27^ in humans. This signifies that tight regulation of GH storage in secretory granules and its subsequent release is absolutely necessary for normal functions of hormone. Furthermore, it was shown that transfection of GH and structurally similar hormone (prolactin, PRL) in AtT20 cells (pituitary cell line) results in their aggregation and secretory granules formation; whereas these hormones do not aggregate when expressed in non-pituitary cells^28^,^29^. The data suggests that optimized cellular conditions might be necessary for aggregation and storage of these pituitary hormones. Interestingly, bovine somatomammotrophs store GH and PRL as separate aggregates within the same granule^30^, suggesting their different mechanism/requirement of storage.

In the current study, we used recombinant GH to study the *in vitro* aggregation and amyloid formation. Analysis of protein sequence with TANGO (aggregation prediction algorithm) showed that GH possesses high sequence specific amyloidogenic potential. Further, using various *in vitro* conditions (such as solvents, glycosaminoglycams (GAGs) and metal ions), GH aggregation was studied. We found that in presence of Zn(II) ions at equimolar concentration, GH formed amyloid-like fibrils. The secondary structural changes due to amyloid formation by GH were monitored by circular dichroism (CD) spectroscopy, which showed reduction in helicity. The GH aggregates were shown to bind amyloid specific dyes such as Congo red (CR) and Thioflavin T (ThT) and displayed curvy fibril morphology under transmission electron microscopy (TEM). The nuclear magnetic resonance (NMR), time-resolved fluorescence and mass spectrometry studies showed that immediately after addition of Zn(II) ions to freshly dissolved GH, the oligomerization of protein was initiated, which eventually favors amyloid formation. The involvement of Zn(II) in amyloid formation is further confirmed as in presence of Zn(II) and ethylenediaminetetraacetic acid (EDTA, metal chelator), GH did not form any amyloid after long incubation. Our data thus provides an insight into the possible mechanism of GH storage and its release from secretory granules of anterior pituitary.

## Results

### Amyloid formation by GH in pituitary tissue

**Figure 1:**
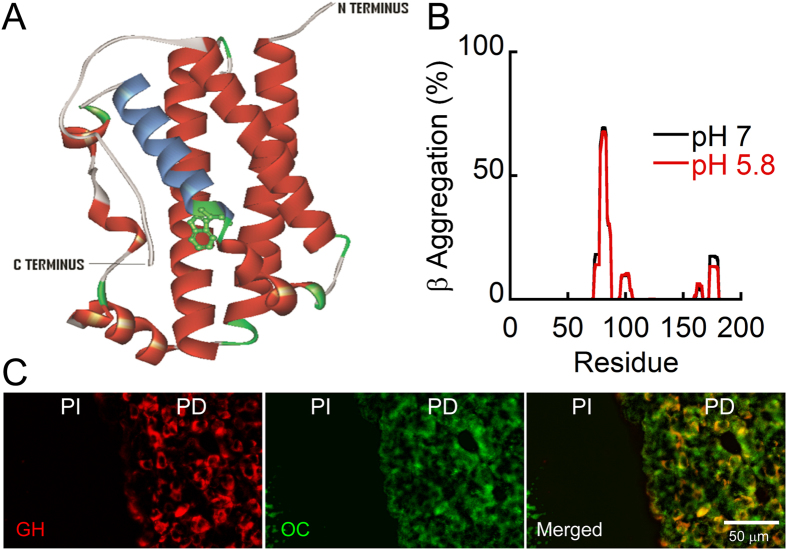
GH is stored as amyloids within rat pituitary tissue. (**A**) Structure of GH displaying its four major helices and a single tryptophan residue (PDB ID: 1HGU). (**B**) TANGO algorithm showing the aggregation prone residues at pH 5.8 and 7.0. The residues 72–82 showed maximum aggregation tendency at both the pHs. (**C**) Immunohistochemistry showing colocalization of amyloid specific antibody OC with GH. OC antibody is shown in green color that colocalized with GH (present in Pars distalis (PD) of anterior pituitary) shown in red color and the right panel shows the merged image where yellowish signal depicts the co-localization of GH and amyloid signals. PI denotes Pars Intermedia of anterior pituitary. Scale bar is 50 μm.

According to the crystal structure of the recombinant GH/receptor complex (3HHR), GH mainly possesses a helical conformation with four major helices^31^,^32^. GH also contains two disulfide bridges, one of which joins distant parts of the molecule (large loop) while the other forms a small loop near the COOH terminus^33^ (Fig. 1A). In order to predict whether any region of this highly helical protein possess an aggregation potential, we performed TANGO analysis^34^ using the amino acid sequence of GH at pH 7.0 and 5.8. The analysis predicted one region (72–82) with high propensity and three other regions with relatively lower propensity (Fig. 1B) of β aggregation at both the pHs. The fibrillation propensity of GH was further evaluated using Zipper DB software^35^, which predicted 4 segments (49–54; QTSLCF, 53–58; CFSESI, 98–103; ANSLVY, 155–170; ALLKNY) as β aggregation “hot spots” (Supplementary Fig. S1). Therefore, the *in silico* analyses clearly reveal that GH possess high propensity to aggregate and is capable to form amyloid under suitable conditions.

To study the storage form of GH in secretory granules of pituitary glands, immunohistochemistry was performed using rat pituitary tissue. Previously, it was shown that GH stored in anterior pituitary binds amyloid specific dye, Thioflavin S (ThioS)^14^. To further ascertain the nature of GH aggregates in rat pituitary, we used immunohistochemical studies of rat pituitary tissue sections using anti-GH antibody and amyloid-specific antibody (OC)^36^. Consistent with previous report^14^, our results show that GH positive cells (stained with anti–GH antibody) present in Pars distalis (PD) of anterior pituitary were highly colocalized with OC staining. The abundant OC staining compared to GH staining could be due to the presence of other protein/peptide hormone amyloids in secretory granules^14^ of pituitary tissue (Fig. 1C).

### Secondary structural transition of GH in various *in vitro* conditions

To test amyloid formation of GH, freshly prepared protein in 5% D-Mannitol, pH 5.5, 0.01% sodium azide was treated with a various *in vitro* conditions that were reported to cause amyloid fibril formation in other proteins. D-Mannitol is a commonly used stabilizing agent in many protein/peptide formulations and has been used as solvent in peptide/protein aggregation studies previously^14^,^37^,^38^,^39^,^40^,^41^,^42^. The conditions used for studying GH aggregation include various percentages of 2,2,2 trifluroethanol (TFE)^43^,^44^, different concentrations of sodium chloride (NaCl)^45^, various GAGs^14^,^40^ and metal ions^46^,^47^. GH was incubated with each of these conditions with slight agitation at 37 °C for 15 days and protein secondary structural transition was analyzed using CD spectroscopy and amyloid formation was detected using ThT binding and CR binding. Further, TEM was done on GH samples incubated in various conditions to analyze the morphology of the aggregates formed.

**Figure 2:**
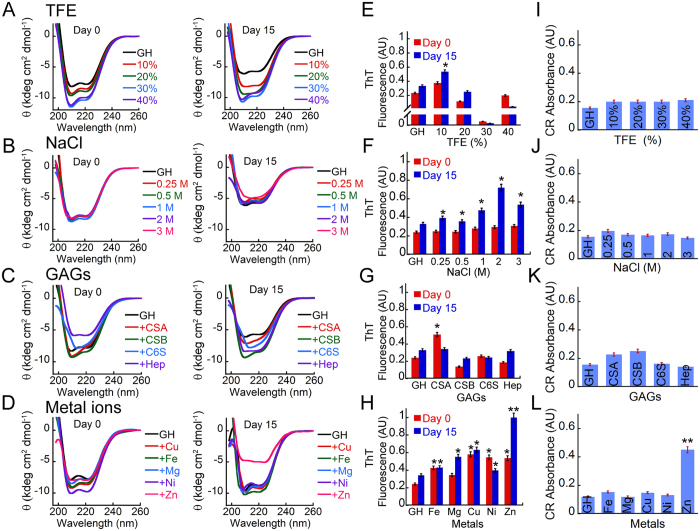
*In vitro* aggregation of GH. (**A**–**D**) Secondary structural transition of GH detected by CD in presence of various incubating conditions such as TFE (0–40%), varying NaCl concentrations (0–3 M), GAGs (CSA, CSB, C6S, and Hep) and various metal ions (Cu(II), Ni(II), Fe(II), Mg(II) and Zn(II)). (**E**–**H**) Amyloid fibril formation of GH probed by ThT fluorescence at 482 nm on day 0 and day 15 showing GH aggregation in presence of various *in vitro* conditions. Statistical significance: 0.05 > *p > 0.01, **p < 0.01 with respect to GH alone at day 0. (**I**–**L**) The amyloid nature of GH aggregates after 15 days of incubation were further verified with CR binding assay. CR absorbances at 540 nm are plotted for each condition. Statistical significance: 0.05 > *p > 0.01, **p < 0.01 with respect to GH alone at day 15. Error bar represents standard error.

The CD studies showed that GH is mostly a helical protein as it showed two distinct minima at ~220 nm and ~208 nm. When GH solutions were mixed with varied percentages of TFE (0–40%), GH showed an increase in negative ellipticity at 222 nm and 208 nm, suggesting stabilization of helical conformation in presence of TFE at day 0 (Fig. 2A). Maximum helicity was observed in presence of 30% TFE. The CD spectra of GH however remained similar in all concentration of TFE even after 15 days of incubation at 37 °C, indicating that no significant structural alteration occurred in presence of TFE within studied time period. Similarly, when GH solutions were treated with different concentrations of NaCl, no significant change of CD spectra was observed for any of the concentration of NaCl studied. The data suggest that NaCl did not induce any structural changes of GH at day 0. However, when these solutions were incubated for 15 days, the secondary structure of GH incubated alone and in presence of various NaCl concentrations (except 3 M NaCl) remained unchanged and were mostly helical in nature. Interestingly, GH incubated in presence 3 M NaCl showed β-sheet like conformation in CD after 15 days (Fig. 2B) of incubation.

We also investigated the effect of secretory granule relevant conditions such as presence of GAGs and metal ions on GH amyloid formation *in vitro*. GAGs are suggested to play an important role in formation and stabilization of amyloid fibrils associated with human diseases^48^,^49^,^50^ as well as packaging of proteins in secretory granules^51^. Recently it was also suggested that many hormones could form amyloid *in vitro* in presence of GAGs like chondroitin sulphate A (CSA) and heparin (Hep)^14^. To study the amyloid formation of GH in presence of various GAGs, GH solutions were incubated with equimolar concentrations of various GAGs (CSA, chondroitin sulphate B (CSB), chondroitin 6 sulphate (C6S) and Hep). CD spectroscopy was performed to monitor the secondary structural changes of the protein in presence and absence of GAGs. Depending upon the GAGs, CD spectrum of GH showed differences in helicity when compared to GH in absence of GAGs (Fig. 2C). GH with CSB showed maximum helicity as suggested by higher minima values at ~222 nm and ~208 nm. Interestingly, GH in presence of C6S showed a single minimum at ~208 nm and the minima at 222 nm was almost absent. GH in presence of CSA showed similar CD spectrum to GH solution in presence of C6S with increased minima at ~208 nm. On the contrary, CD spectrum of GH in presence of heparin showed a decrease in helicity. After 15 days of incubation, no significant changes in CD spectra were observed in presence of different GAGs indicating that the secondary structure observed at day 0 was stable even after 15 days of incubation. In few cases, only decrease in CD signal was observed, which might be due to the aggregation and/or precipitation of the protein.

Divalent cations are usually present in trans-Golgi and are reported to play an important role in regulating and inducing the protein aggregation destined for secretory granule formation^52^. To study the aggregation of GH in presence of various metal ions, 500 μM freshly prepared GH in 5% D-Mannitol with 0.01% azide, was incubated with equimolar concentrations of various metal ions (CuSO_4,_ NiSO_4,_ FeSO_4,_ MgSO_4_ and ZnSO_4_) and aggregation of GH was monitored as mentioned previously. CD data indicates that, immediately after addition of metal ions, GH remained mostly in helical conformation; although subtle increase in molar ellipticity was observed in presence of Ni(II) and Zn(II) ions. After 15 days of incubation, the helicity of GH remained same both in presence and absence of metal ions (Fig. 2D), except in presence of Zn(II) and Ni(II) ions. GH in presence of Zn(II) and Ni(II) showed significant reduction of helicity. The data suggested that among all the metal ions used in this study, Zn(II) and Ni(II) altered the GH conformation after 15 days of incubation.

### Monitoring the amyloid formation by ThT fluorescence and CR binding

ThT is a fluorescent dye that is frequently used for monitoring the amyloid formation by proteins and peptides^53^,^54^. The emission intensity of ThT dye at 480 nm (excitation at 450 nm) increases significantly when it binds to cross β-sheet structure of amyloids. Along with secondary structural transition monitored by CD spectroscopy, ThT binding study was also performed for monitoring the amyloid formation by GH. The data showed that immediately after addition of 10% TFE, an increase in ThT fluorescence at 482 nm was observed (Fig. 2E) compared to GH alone sample. In presence of 20% TFE, a significant reduction of ThT fluorescence was observed. In presence of 30% and 40% TFE, ThT fluorescence was less compared to GH alone sample at day 0. After 15 days of incubation, GH in presence of 10% and 20% TFE showed an increase in ThT fluorescence, suggesting the possible formation of aggregated structures. However in 30% and 40% TFE, a significant reduction in ThT fluorescence was observed (Fig. 2E). When GH solution was mixed with various concentration of NaCl, most of the GH solution showed subtle increase in ThT binding on the initial day (day 0). After 15 days of incubation, GH in presence of all concentrations of NaCl showed increase in ThT fluorescence. However, significant ThT binding was only observed for 2 and 3 M of NaCl (Fig. 2F). GH in presence of CSA showed an increase in ThT binding at day 0; however, after 15 days of incubation the ThT binding was similar to GH control. The other GAGs did not show any significant binding both at day 0 and after 15 days of incubation, suggesting GAGs do not favor any amyloid like aggregation of GH under the solution conditions of this study (Fig. 2G). The GH solutions incubated with metal ions showed varied response for ThT fluorescence. GH in presence of all metal ions showed slight increase in ThT fluorescence compared to GH alone sample at day 0. After 15 days of incubation, GH in presence of Cu(II), Mg(II) and Zn(II) showed increase in ThT binding. Among these, highest ThT binding was observed for GH in presence of Zn(II). The GH incubated in presence of Ni(II) showed subtle reduction in ThT fluorescence after 15 days of incubation (Fig. 2H).

To investigate the amyloidogenic nature of various GH aggregates formed in different conditions, we further performed CR binding assay. CR is a dye routinely used to detect β-sheet–rich assemblies of amyloid^55^. CR binding is generally measured by the “red shift” of its absorption maxima from 490 to 540 nm and by an increase in the dye’s molar absorbance. The data showed that incubated GH in all conditions displayed CR binding similar to the GH alone sample, except GH incubated in presence of Zn(II). In all these samples, (except incubated sample in presence of Zn(II)), the CR absorption spectra neither showed characteristic red shift nor any significant increase in CR absorptivity (Fig. 2I–L and Supplementary Fig. S2). The GH aggregate formed in presence of Zn(II) ions however showed the characteristic red shift upon CR binding as well as increase in molar absorbance (Fig. 2L) indicating the amyloidogenic nature of these aggregates.

### Morphological characterization of GH aggregates

**Figure 3:**
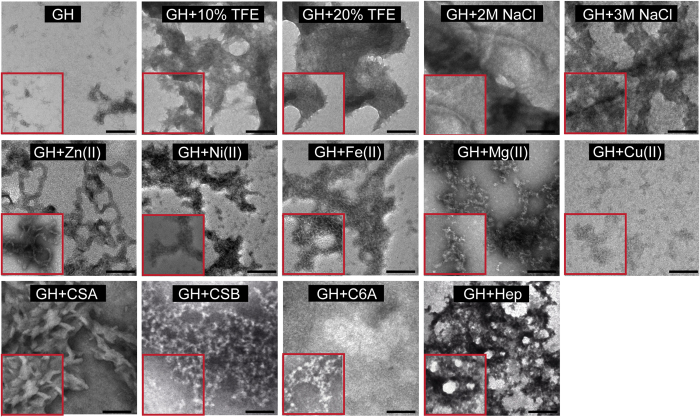
Morphology of GH aggregates. Electron micrographs showing GH aggregates formed in presence of various aggregating conditions. GH aggregates in presence of 2 M and 3 M NaCl and CSA showed some ordered aggregates, whereas the rest of the conditions showed mostly amorphous aggregates (except GH samples incubated with metals). GH in presence of Zn(II) showed short curvy fibrils, whereas other metals induce small oligomeric structures. Scale bar are 200 nm. The enlarged image (inset) showing the closer view of aggregate morphology.

To analyze the morphology of the GH aggregates, TEM analysis was performed with those samples, which showed maximum ThT binding after 15 days of incubation. GH in presence of 10% and 20% TFE showed mostly amorphous aggregates similar to incubated GH alone sample. Whereas GH in presence of 2 M and 3 M NaCl showed some ordered aggregates along with amorphous aggregates (Fig. 3). The morphological study of GH incubated in presence of equimolar concentrations of Cu(II), Ni(II), Fe(II) and Mg(II) ions showed mostly oligomeric aggregates of varying size (Fig. 3). In contrast, GH incubated in presence of Zn(II) for 15 days, displayed short curvy filamentous morphology (Fig. 3) similar to fibrils observed in other amyloidogenic proteins^56^,^57^,^58^. The data suggests that GH can attain fibril morphology only in presence of Zn(II) ions. Further, the GH aggregates formed in presence of various GAGs showed mostly amorphous aggregates (Fig. 3). Interestingly, in presence of CSA, GH showed some ordered aggregates under TEM. However, the low ThT and CR binding of this aggregates (Fig. 2G,K) precludes the possibility of amyloid formation.

### Characterization of GH aggregates formed in presence of Zn(II) ions

**Figure 4:**
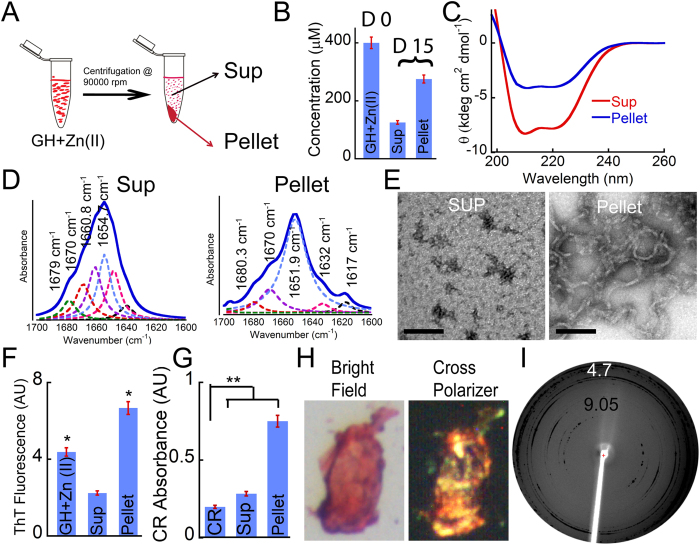
Characterization of GH aggregates formed in presence of Zn(II). (**A**) Schematic representation of isolating GH aggregates formed in presence of Zn(II). The aggregate formed after 15 days of incubation was centrifuged at 90,000 r.p.m for 60 mins to obtain supernatant (Sup) and pellet fraction. (**B**) Concentration measurement by UV absorbance of Sup revealed that more than half (225 μM) of GH monomer aggregated into pelletable fibrils. (**C**) CD spectra of Sup and pellet fraction showing helical conformation. (**D**) The supernatant and pellet fractions were analyzed with FTIR. Deconvoluted FTIR spectra of GH from Sup and pellet fractions showing helix as a major secondary conformation (~1656 cm^−1^). The colored dotted lines in the spectra of both Sup and pellet depicting the Lorentzian curve fit spectra of the individual peak. GH aggregates formed in presence of Zn(II) showing two additional peaks at 1632 and 1617 cm^−1^ represents the β sheet content of GH fibrils. (**E**) The morphology of the aggregates observed under TEM showing small amorphous aggregates in Sup and short curvy fibrils in pellet, respectively. Scale bar: 100 nm. (**F**) ThT binding of both Sup and pellet fractions. The pellet fraction showed higher ThT fluorescence than Sup. Statistical significance, 0.05 > *p > 0.01, **p < 0.01 with respect to GH alone at day 0. (**G**) CR absorbance at 540 nm of both pellet and Sup fractions. The pellet fraction showed higher CR absorbance than Sup. Statistical significance, 0.05 > *p > 0.01, **p < 0.01. (**H**) The greenish-yellow CR birefringence of pellet under crossed polarized light indicates the amyloid formation by GH. The corresponding bright-field image is also shown in the left panel. (**I**) X-ray diffraction pattern of GH fibril formed in presence of Zn(II) showing reflections at 4.7 and 9 Å suggesting the amyloid nature of these fibrils. Error bar represents standard error.

Among the various aggregating conditions under this study, GH aggregates in presence of Zn(II) only showed amyloid like characteristics as these aggregates showed high ThT/CR binding and fibrillar morphology under TEM (Table 1). In this context, previous reports also suggested that Zn(II) plays a significant role in aggregation and secretory granule formation of GH^29^,^59^. Therefore, we decided to investigate the aggregation of GH in presence of Zn(II) in more detail. To study this, 500 μl of freshly prepared GH solution (400 μM) was incubated in presence and absence of 400 μM Zn(II) for 15 days. After fibril formation was confirmed by TEM analysis, 100 μl aliquots of this solution were taken for ultracentrifugation (90,000 r.p.m, 60 mins) to isolate fibrils from soluble GH (Fig. 4A). The amount of aggregate formed in presence of Zn(II) was calculated from the GH concentration present in supernatant; measured using UV absorption method. The data revealed that from the 400 μM GH monomer incubated for 15 days in presence of Zn(II), 175 μM GH remained in soluble form and rest of the GH monomer got incorporated into fibrils (225 μM) (Fig. 4B). The conformation of GH in the supernatant and pellet was studied by CD and Fourier transform infrared (FTIR) spectroscopy. CD spectra revealed helical conformation for both supernatant and pellet fraction with varying degree of helicity as observed before (Figs 4C and 2D). The FTIR spectra in the range of 1600 cm^−1^ to 1700 cm^−1^ (amide-I band) were deconvoluted and curve fitted for the analysis of protein secondary structure^60^. The FTIR spectra of both supernatant and pellet showed a major absorbance peak at 1654 and 1651 cm^−1^ indicating the presence of mostly helical structure (Fig. 4D). The FTIR spectra of GH pellet however showed additional peaks at 1632 and 1617 cm^−1^ indicative of the presence of β-sheet in the sample^61^,^62^. Further, the morphology of the supernatant and pellet was studied using TEM, wherein the pellet revealed the presence of curvy fibrils (Fig. 4E) and the supernatant showed mostly small amorphous aggregates (Fig. 4E). Further, the ThT fluorescence study of both supernatant and pellet was performed, which revealed a higher ThT binding of GH in pellet fraction (Fig. 4F) compared to GH in soluble fraction. To ascertain the amyloid nature of the GH aggregate formed in presence of Zn(II), CR binding using three different techniques (CR absorbance, CR fluorescence and CR birefringence) were performed using GH in pellet fractions. The addition of CR to pellet fraction increased the molar absorbance of CR (Fig. 4G) and also showed an increase in CR fluorescence (Supplementary Fig. S3). Further, when isolated GH aggregates were treated with alkaline CR and observed under a polarizing microscope, the aggregates showed green gold birefringence under cross-polarized light (Fig. 4H), suggesting GH formed amyloid like aggregates in presence of zinc. Finally using X–ray diffraction techniques, GH fibrils were tested for the presence of characteristic diffraction pattern found in amyloid fibrils. The cross-β-sheet of amyloid fibrils generally shows two reflections; meridional at 4.7 Å, which corresponds to the distance between two β-strands and a variable equatorial reflection at ~10 Å, which generally represents the distance between two β-sheets in a cross-β-sheet motif of amyloid fibrils^63^. GH fibrils formed in presence of Zn(II) showed diffractions at 4.7 Å and at 9.05 Å (Fig. 4I) suggesting presence of cross-β-sheet structure, as seen in amyloid fibrils^64^.

### Zinc initiated oligomerization of GH

**Figure 5:**
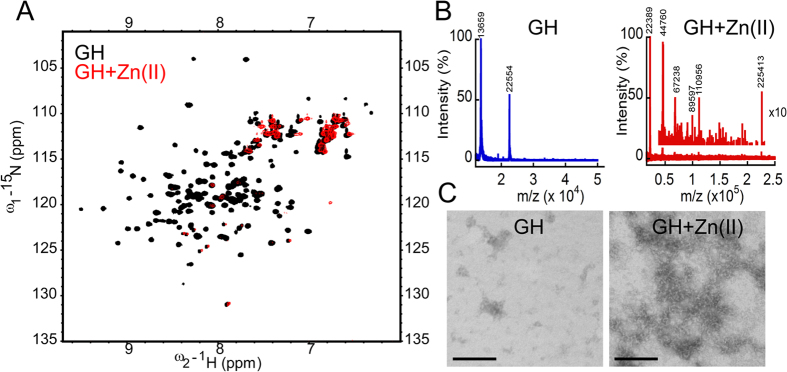
Zn(II) ions initiate oligomerization of GH monomers. (**A**) HSQC NMR spectrum of freshly dissolved GH in absence (black) and presence (red) of Zn(II) showing immediate oligomerization of GH in presence of Zn(II). (**B**) MALDI TOF of GH in absence (blue) and presence (red) of Zn(II) showing GH oligomers in presence of Zn(II). Inset is showing the magnified (10X) oligomer peaks. Freshly dissolved GH showed major peak corresponding to the molecular weight of monomer. (**C**) Morphology of GH incubated in presence and absence of Zn(II) was analyzed using TEM showing GH oligomer formation in presence of Zn(II). Scale bar: 200 nm.

To study the interaction of Zn(II) with GH and the possible mechanism of Zn(II) mediated amyloid aggregation, we performed two dimensional NMR, mass spectrometry and time resolved fluorescence study in presence and absence of Zn(II). The NMR technique of Heteronuclear Single Quantum Correlation (HSQC) study using N^15^ labeled protein revealed well-dispersed peaks (Fig. 5A) indicating the defined structure of GH in solution, which is consistent with our CD data. When equimolar concentration Zn(II) was added to the GH solution, most of the NMR peaks disappeared and only certain side chain peaks were visible. The data suggest an immediate oligomerization of protein upon addition of Zn(II) (Fig. 5A). Further, matrix-assisted laser desorption/ionization (MALDI) time of flight (TOF) mass spectrometry of GH in presence of Zn(II) showed many oligomeric peaks from dimer to dodecamer (225 kDa), whereas GH alone showed mostly monomeric peak (Fig. 5B). Furthermore, morphological analysis using TEM of freshly dissolved GH solution showed presence of small amorphous aggregates, whereas, immediately after addition of Zn(II) in protein solution, GH transformed into large oligomeric species (Fig. 5C). Although, we are not certain at this point whether these oligomers directly transformed into fibrils, however, all these data clearly indicate that zinc induces immediate oligomerization of GH that facilitates its amyloid formation *in vitro*. In order to analyze whether GH oligomers formed in presence of Zn(II) have exposed hydrophobic surfaces (typical property of amyloid oligomers^65^,^66^,^67^,^68^), we performed 8-Anilinonaphthalene-1-sulfonic acid (ANS) binding assay. ANS spectra of GH in presence and absence of Zn(II) were recorded by exciting the sample at 370 nm and emission was recorded in the range of 400–600 nm. While the GH alone showed ANS emission maxima (λ_max_) at 500 nm, the GH oligomers formed in presence of Zn(II) showed an increased ANS fluorescence intensity with blue shift of λ_max_ to ~20 nm. The data indicates high ANS binding of GH oligomers (due to exposed hydrophobic surfaces) formed in presence of Zn(II) (Supplementary Fig. S4).

**Figure 6:**
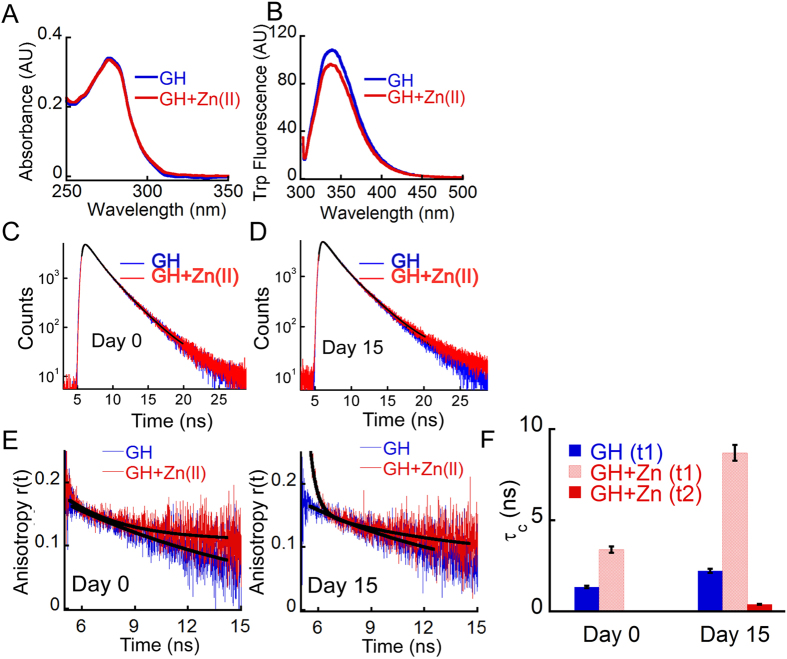
Time resolved fluorescence intensity and anisotropy decay study of GH oligomerization. (**A**) Absorbance spectra of freshly prepared GH in absence and presence of Zn(II) showing similar UV absorbance. (**B**) Emission spectra of the GH in presence and absence of Zn(II) showing a decrease in Trp fluorescence intensity in presence of Zn(II). (**C**,**D**) Time resolved Trp fluorescence intensity decay of GH in presence and absence of Zn(II) at day 0 and day15 indicating negligible change in Trp microenvironment after amyloid fibril formation. (**E**) Time resolved anisotropic decay and (**F**) rotational correlation time of GH in presence and absence of zinc indicating that GH monomer on day 0 (left) possess single exponential decay kinetics with sub-nanosecond rotational correlation time. In presence of zinc, GH sample showed longer rotational correlation time at day 0 indicating immediate oligomerization. After 15 days of incubation (E, right) the two components (τ1 and τ2) indicating an increase in overall size of the protein due to fibril formation. Error bar represents standard error.

To further study the oligomerization of GH in presence of Zn(II), time-resolved fluorescence intensity and anisotropic decay study was performed. GH contains one Trp residue at 86^th^ position, therefore time resolved fluorescence intensity and anisotropy decay kinetics can provide local as well as global structural changes due to Zn(II) induced oligomerization. The absorption spectra of freshly prepared GH solution both in presence and absence of Zn(II) showed characteristic tryptophan absorption spectra with maxima around 280 nm (Fig. 6A). Further, protein solutions were excited at 280 nm and the fluorescence spectra was recorded in the range of 300–500 nm. The fluorescence λ_max_ was observed at 340 nm for both GH alone sample and GH in presence of Zn(II). Moreover, we also observed a decrease in overall fluorescence intensity of GH sample in presence of Zn(II) (Fig. 6B). This decrease in fluorescence intensity of GH in presence of Zn(II) could be due to oligomerization, where Trp fluorescence got quenched due to the near vicinity of quencher amino acids and/or partial precipitation of the protein. Interestingly, the fluorescence lifetime of GH in presence and absence of Zn(II) at day 0 was identical, which remained unaltered during the course of aggregation and amyloid formation even after 15 days of incubation in presence of Zn(II) (Fig. 6C,D). The data suggests that the local environment of Trp in GH was mostly unaltered during fibril formation.

The time-resolved fluorescence anisotropy decay of GH on day 0 and day 15 showed single exponential decay with sub-nanosecond rotational correlation time (τ_c_). A small initial anisotropy (~0.2 ns) suggests the presence of a fast segmental motion (Fig. 6E). However, immediately after addition of Zn(II) ions in GH solution, the anisotropy decay was still single exponential but the rotational correlation time was comparatively higher than GH alone sample (Fig. 6E,F), indicating oligomerization of GH upon addition of zinc. The data was consistent with our NMR and MALDI experiments (Fig. 5A,B). After 15 days of incubation, GH in presence of zinc showed two exponential anisotropy decays, where a longer correlation time (~8.7 ns) was observed along with an emergence of a short component (~390 ps). This increase in rotational correlation time might be due to the formation of large GH aggregates in presence of Zn(II) after 15 days of incubation. The appearance of a smaller correlation time could be due to the altered segmental motion around Trp residue of GH after fibril formation (Fig. 6F). In contrast, the GH alone sample did not show any significant difference in rotational correlation time (τ_c_) after 15 days of incubation.

### GH is co-stored with Zn(II) in anterior pituitary

**Figure 7:**
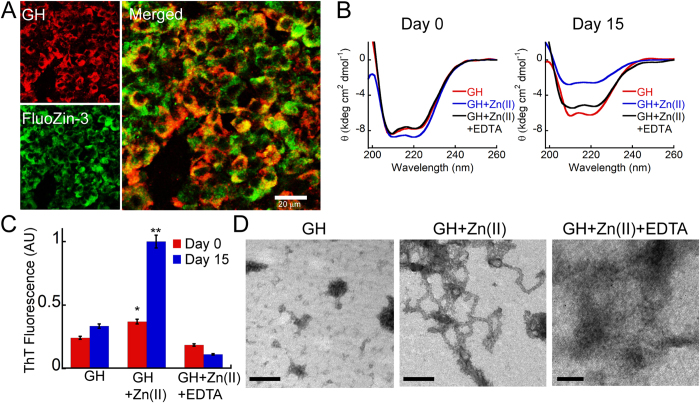
Colocalization of GH and Zn(II) in pituitary tissue. (**A**) Immunostaining of rat pituitary tissue performed using Zn(II) detecting dye FluoZin-3 and anti-GH antibody showing colocalization (yellow in overlay image). The anti-GH antibody is shown in red and FluoZin-3 dye is shown in green. Scale bar is 20 μm. (**B**) CD spectrum of GH samples incubated in presence of Zn(II) and Zn(II) + EDTA at day 0 and day 15. On day 15, GH incubated in presence of Zn(II) showing higher reduction in helicity compared to GH incubated in presence of Zn(II) + EDTA and GH alone sample. (**C**) ThT fluorescence showing higher ThT binding for GH incubated in presence of zinc ions. GH alone sample was used as control. GH in presence of Zn(II) and EDTA (metal chelating agent) showing decrease in ThT binding. (**D**) TEM of GH incubated in presence of Zn(II) showed short curvy fibrils, whereas GH in presence of Zn(II) + EDTA showed mostly amorphous aggregates indicating the importance of Zn(II) in GH amyloid formation. Scale bar: 200 nm. Error bar represents standard error. Statistical significance, 0.05 > *p > 0.01, **p < 0.01 with respect to GH alone at day 0.

To demonstrate the role of Zn(II) ions in GH secretory granules *in vivo*, rat pituitary tissue was subjected to immunostaining with anti-GH antibody and FluoZin-3, which binds and detects zinc in cells and neuronal tissues^69^,^70^. Our results showed abundant FluoZin-3 staining in pituitary compared to the anti-GH antibody staining in pituitary. This is because other than somatotrophs, zinc is also reported to be present in cells such as corticotrophs and thyrotrophs in rat anterior pituitary^71^,^72^. However, there is significant colocalization between FluoZin-3 and anti-GH antibody staining suggesting a possible contribution of Zn(II) ions for aggregating GH in secretory granules of rat pituitary (Fig. 7A). Since our study also shows that GH is stored in amyloid-like structure in tissues (Fig. 1C), Zn(II) ions may be additionally proposed to play an important role in amyloid-like aggregation of GH for their storage in secretory granules. Furthermore, incubation of GH monomers with Zn(II) forms short fibrils with amyloid like tinctorial properties; however GH monomer incubated with both Zn(II) and EDTA for 15 days *in vitro* neither bind ThT nor showed fibril morphology under TEM (Fig. 7B–D). These data therefore demonstrate that Zn(II) ions co-stored with GH inside secretory granules of pituitary tissue, might play an important role in GH aggregation/amyloid formation.

### Monomer release by GH amyloids

**Figure 8:**
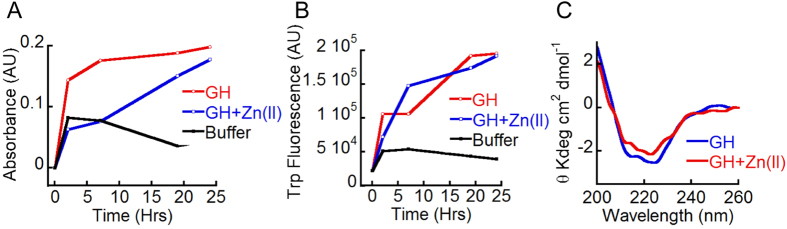
Monomer release from GH amyloid fibrils. The isolated GH fibrils formed in presence of Zn(II) were redissolved and the solution was dialyzed using a 50 KDa MWCO dialysis membrane against 10 mM Tris HCl, pH 7.4. (**A**) Increment in absorbance at 280 nm of solution outside the dialysis membrane denoting release of monomers. GH incubated alone for 15 days and 5% D-Mannitol solution were also dialyzed against buffer as controls. (**B**) Trp fluorescence outside the dialysis membrane showing increase with time indicating release of GH monomers. (**C**) CD spectrum of released monomers showing helical conformation of the protein.

To demonstrate, that the GH fibrils formed in presence of Zn(II) ions are capable of releasing monomers, 500 μl of GH (400 μM) in presence of equimolar concentration of Zn(II) was incubated for 15 days until fibril formation was observed by TEM. 100 μl aliquots of this incubated solution were centrifuged (90,000 r.p.m, 60 mins) to isolate the fibrils. The isolated fibrils were then redissolved in 100 μL of 5% D-Mannitol, pH 5.5 and were dialyzed against 10 mM Tris HCl, pH 7.4, using a 50 kDa molecular weight cut off (MWCO) dialysis membrane. The use of 50 kDa MWCO was to ensure that the proteins released outside are mostly monomers and dimers. During dialysis, we measured the UV absorbance at 280 nm and Trp fluorescence at 340 nm at regular intervals. The UV absorption and Trp fluorescence values at 280 nm and 340 nm, respectively for released GH were plotted against dialysis time. Our data showed an increase in absorbance at 280 nm (Fig. 8A) and Trp fluorescence intensity (Fig. 8B) during dialysis. Further, CD analysis of the aliquot showed a helical conformation suggesting that released GH retained its native secondary structure (Fig. 8C). GH incubated alone for 15 days as well as 5% D-Mannitol solution were used as controls in this assay. The latter control was kept to ensure that the chemical and/or any contamination of membrane do not interfere with the release assay. Our data suggest that upon dilution, GH amyloid is able to release monomers.

## Discussion

Understanding protein/peptide aggregation into amyloid formation is important as this is associated with two dozen human diseases such as Alzheimer’s and also with many native biological functions^1^,^3^ including melanosome biogenesis^5^,^6^ and secretory granule biogenesis^14^. It was known for a long time that protein/peptide destined to regulated secretion are required to aggregate in trans-Golgi for secretory granule formation^29^,^73^. Previous studies suggested that various factors such as low pH^74^, availability of helper molecules such as biogenic amines^75^, GAGs^39^,^51^,^76^ and helper protein such as chromogranins^77^ are actively involved in secretory granule biogenesis of protein/peptide hormones. It was recently suggested that protein/peptide hormones are stored inside the secretory granules as amyloid-like structures^14^. It was also shown that not all hormones formed amyloid fibrils *in vitro* under identical conditions. For example, although hormone likes corticotrophin releasing factor, Galanin, β-endorphin forms amyloid at low pH and in the presence of heparin, human PRL requires CSA for its amyloid formation *in vitro*^14^. This specific requirements might be essential for controlling the formation of individual granules, the content of the granules and also the subsequent release of the contents. In our current study, we carried out biophysical investigation of GH aggregation and amyloid formation relevant to its storage in secretory granule of pituitary. The immuno fluorescence co-localization study showed that antibody staining of GH co-localizes highly with amyloid specific OC antibody staining suggesting the presence of amyloid-like state of GH in the secretory granules of rat pituitary (Fig. 1C). The present data is also consistent with the previous study using the amyloid-staining agent Thio S, which showed GH could form amyloid *in vivo*^14^.

To study the aggregation and amyloid formation of GH *in vitro*, we used various solution conditions that were used previously for studying amyloid formation by proteins, associated with human diseases and secretory granule biogenesis^14^,^40^,^46^,^48^,^52^,^56^. We found that GH aggregation occurs in many experimental conditions, however, these aggregates did not fulfill all the characteristics of amyloids (Table 1). For example, when GH was incubated in presence of CSA, ordered aggregates were seen under TEM (Fig. 3). However these aggregates did not show any ThT and CR binding (Fig. 2G,K). The data suggests GH is unable to form amyloid like aggregates in presence of CSA. Further, GH also failed to show amyloid-like aggregates in presence of various other GAGs under the experimental conditions used in this study (Fig. 2C,G,K). Interestingly, PRL, another lactogenic hormone found in anterior pituitary, which has structural similarity to GH^78^ was shown to form amyloid-like fibrils in presence of CSA^14^. The data suggests that, irrespective of structural similarity of GH and PRL^78^ and their similar storage location in anterior pituitary, both proteins may require different helper molecules to form amyloid like aggregates. The data further confirm the role of specific helper molecules in the selection and packaging of various protein/peptide hormones in secretory granules formation^14^,^39^,^40^,^79^.

Previous studies have shown that metal ions facilitate the aggregation of protein/peptide hormones for secretory granule biogenesis^52^. Moreover several previous studies suggested that Zn(II) plays a crucial role in GH secretory granule biogenesis^29^,^72^,^80^,^81^. In this context, when GH aggregation was studied in presence of various metal ions, GH aggregates formed in presence of Zn(II) only showed increased ThT fluorescence (Figs 2H and 4F), significant CR binding (Figs 2L and 4G–H) and fibrillar morphology when studied under TEM (Figs 3 and 4E). In contrast to Zn(II), other metal ions however did not induce any amyloid formation of GH (Figs 2H,L and 3). While studying the mechanism of Zn(II) mediated GH aggregation, we found that Zn(II) immediately induced the oligomerization of protein as suggested by NMR, mass spectrometry and electron microscopy study (Fig. 5). However, the GH oligomerization due to Zn(II) addition did not induce any major structural changes as indicated by the time resolved Trp fluorescence (Fig. 6C) and CD spectroscopy at day 0 (Fig. 2D). However, we are not certain at this point whether these oligomers are directly converting to fibrils during incubation (because these oligomers could be off-pathway oligomers^82^,^83^,^84^). However our data clearly indicates that rapid oligomerization of GH in presence of Zn(II) is essential for GH fibrillation, which may eventually facilitate GH storage within secretory granules. In consistent with this observation, Zn(II) is also reported to be present in trans-Golgi and within the secretory granules of various neuroendocrine cells^71^. Our study also suggests that Zn(II) may participate in secretory granule formation, by assisting in protein aggregation, and also gets co-stored within the secretory granules of GH. Further, in trans-Golgi, Zn(II) could mediate rapid oligomerization of GH, which enables it’s sorting into the regulatory secretory pathway^71^,^81^. Previous reports have shown that GH binds to Zn(II) with high affinity using its amino acid side chain of H18, H21 and E74, which results in dimerization of GH^29^,^72^. Moreover in the secretory granules, the total amount of Zn(II) and GH was estimated to be 2 to 5 mM^71^,^72^. Thus, it is quite possible that along with the GH, the major component in GH secretory granules in pituitary is Zn(II). Our immunofluorescence study showed that GH (stained with anti-GH antibody) highly colocalizes with anti-amyloid fibril OC antibody (Fig. 1C) and Zn(II) specific dye FluoZin-3 (Fig. 7A) in anterior pituitary of rat, further supporting the role of Zn(II) in GH aggregation/ amyloid formation within secretory granules. Other than neuroendocrine glands, Zn(II) is also reported to be found in secretory granules of β cells of pancreas and salivary glands, where it facilitates the condensation of insulin and 7 S nerve growth factor (NGF) for their storage^85^,^86^,^87^.

Another interesting observation in our study is that GH forms amyloid in presence of Zn(II) without any major structural changes in the protein as observed by CD and FTIR spectroscopy (Fig. 4C,D). After aggregation and amyloid formation, GH showed only decrease in helicity when studied using CD. Similar observation was also seen before for PRL aggregation and amyloid formation, where the protein did not show any α-helix to β-sheet transition during amyloid formation^14^. In this context, Dobson and coworkers previously reported that amyloid formation by protein cytochrome was accompanied by reduction of helical signal in CD without large conformational transition to β-sheet^88^, which is similar to our observation for GH^88^. Interesting to note that, in contrast to GH and PRL amyloid formation, most natively unstructured^89^,^90^ and/or helical proteins^91^,^92^ undergoes large structural transition to β-sheet (as characterized by a ~218 nm minima in CD spectra) during their amyloid formation. This large conformational transition could be due to the participation of a large segment of protein in cross-β-sheet structure of amyloid. In case of GH, we propose that only a small segment(s) of GH may take part in amyloid fibril formation under this experimental condition, which is consistent with TANGO and Zipper DB analysis (Fig. 1B and Supplementary Fig. S1). Since GH is a highly helical protein with 4 major helices (Fig. 1A), after amyloid formation by small segments, the protein shows mostly a helical conformation in CD. In fact, when the GH aggregates formed in presence of Zn(II) were characterized by FTIR, we found that along with prominent helical content, additional β-sheet signals were also observed (Fig. 4D). This small β-sheet characteristics FTIR peak could be due to participation of small segment of protein in amyloid form (Fig. 4D). Further, the Trp fluorescence analysis (intensity and anisotropy decay kinetics) of Zn(II) induced GH aggregation suggests that the local environment of Trp residue on day 0 and after fibril formation did not change significantly (Fig. 6C–E). The data further indicates that GH does not require major secondary structural changes for its fibrillation. Consistent with this, it has been shown that protein/peptide could form amyloids without major changes in gross conformation of protein but through the involvement of small segment of protein in cross-β-sheet rich amyloid. Therefore, in this situation the protein might be able to maintain most of its globular fold^14^,^93^,^94^,^95^ even after fibril formation.

The most important criteria for an amyloid to be an *in vivo* storage state inside the secretory granules is that amyloid has to be reversible in nature, which may facilitate the release of protein/peptide monomers in functional conformation. Interestingly, GH fibrils formed in presence of Zn(II) are able to release monomers with mostly helical conformation (Fig. 8). The proposed involvement of small segment(s) of GH in amyloid fibril formation could also explain both its efficient amyloid fibril formation *in vivo* (without major mis-folding of the protein into aggregated β-sheet rich structure) and its subsequent release (without requirement of large conformational change of β-sheet to helix) to attain its native functional form.

**Figure 9:**
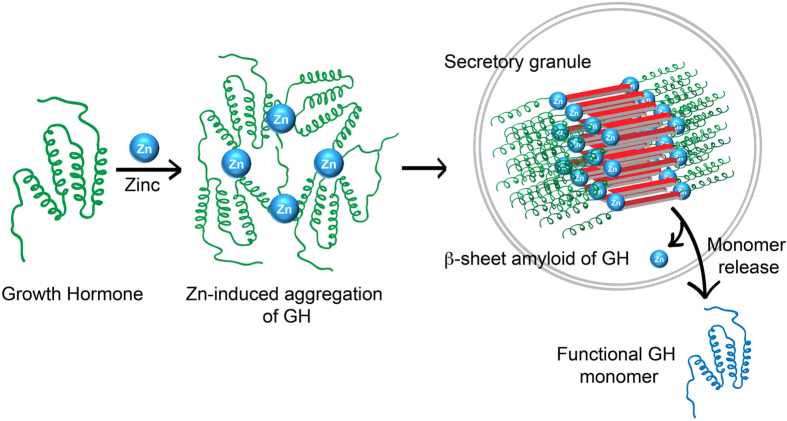
Schematic representation of GH secretory granule formation and monomer release. GH in presence of Zn(II) ion may oligomerize instantly, which may initiate the aggregation and amyloid formation of GH. The GH amyloid subsequently may be sorted and destined for secretory granules biogenesis in somatotrophs of the anterior pituitary. This amyloid can release monomer during secretion along with Zn(II) ions extracellularly.

In summary, the present study suggests that Zn(II) can induce immediate oligomerization of GH, which may facilitate GH aggregation and amyloid formation, and the resultant fibrils are able to release monomers in suitable condition (Fig. 9). The present work therefore highlights the possible mechanism of GH aggregation in presence of Zn(II) and its association with secretory granules biogenesis *in vivo.*

## Materials and Methods

### Chemicals and reagents

Chemicals were obtained from Sigma Chemical Co. (St. Louis, MO) and were of the highest purity available. Water was double distilled and deionized using a Milli-Q system (Millipore Corp., Bedford, MA).

### Expression and purification of human hGH

GH was expressed in *Escherichia coli* BL21 (DE 3) according to the protocol reported^52^,^96^ with slight modification. The human GH plasmid was a kind gift of Prof. Dannies and Prof. Hodsdon from Yale University. Briefly, *E. coli* cells were transformed with pT7L plasmid containing human GH gene and was grown in either terrific broth or M9 media with N^15^ labeled ammonium chloride (for N^15^ labeled protein) till the OD reached 1 at 600 nm. Further, the culture was induced with 1 mM IPTG for 4 hours (hrs) and cells were pelleted at 8,000 r.p.m. for 30 mins. The bacterial pellet was resuspended in 20 mM Tris HCl, pH 8.5 with protease inhibitor cocktail tablet (Roche) and was lysed for 15 minutes sonication on ice. From the cell lysate, inclusion bodies were recovered by centrifugation at 14,000 r.p.m. and were washed twice with 0.5% triton X-100. A solution of 8 M Urea and 2% (v/v) β mercaptoethanol was used to dissolve and denature the pellet. The resulting solution was dialyzed against 20 mM Tris HCl pH 8.5 at 4 °C to refold the protein by slowly removing urea and β mercaptoethanol. After dialysis, the protein solution was centrifuged at 14,000 r.p.m for 1 hr and the supernatant was applied to an ion exchange column (Resource Q, GE healthcare, USA) connected and operated through AKTA purifier FPLC system (GE healthcare, USA). GH bound to the column was eluted with 0.5 M NaCl gradient in 20 mM Tris HCl, pH 8.5 buffer. The major peak of GH from this column was monomeric as assessed by size exclusion chromatography (Superdex 200, GE). Purity of the protein was checked using SDS-PAGE and MALDI TOF analysis. The secondary structure of the pure protein was analyzed by CD spectroscopy and was found to be helical, which confirm the native structure of GH. The monomeric fractions were immediately frozen in liquid nitrogen and lyophilized. The lyophilized protein powder was stored in −80 °C. Solid protein was dissolved in aggregation buffer and the concentration was measured by absorbance at 280 nm using Jasco V-650 spectrophotometer considering the molar ellipticity value of the protein as 17670 M^−1^ cm^−1^.

### Determining GH aggregation propensity

Human GH sequence was subjected to TANGO analysis to detect regions of β aggregation propensity. TANGO calculates aggregation propensity based on simple physico-chemical principles of folding and secondary structure formation of short stretch of amino acids, where it is assumed that aggregating sequence in proteins are fully buried^34^. TANGO for GH sequence was performed at two different pHs 7.0 and 5.0 at 298 K at an ionic strength of 0.02. Further, Zipper DB^35^ was also used to predict “hot spots” of β-aggregation in GH.

### Immunohistochemistry

Adult, male, Sprague-Dawley rats (200–250 g) were used for *in vivo* characterization of secretory granules of GH. Animals were maintained under standard environmental conditions (12 hr: 12 hr, light:darkness cycle, chow and water *ad libitum*). All experimental protocols were reviewed and approved by the Institutional Animal Ethical Committee (IAEC) at NISER, Bhubaneswar, India. Animals were anaesthetized with sodium pentobarbital and perfused transcardially with 50 ml of 10 mM phosphate buffered saline (PBS) pH 7.4, followed by 50 ml 4% paraformaldehyde (PFA) in 100 mM phosphate buffer pH 7.4. Pituitary glands were dissected out and post fixed with 4% PFA overnight at 4 °C. Using 25% of sucrose solution in PBS, the glands were cryoprotected overnight at 4 °C and mounted using tissue tek and sectioned on cryostat (Leica, CM3050S) in coronal plane at 12 μm thickness. Sections were then mounted on poly-L-lysine (Sigma) coated glass slides and stored at −20 °C until processed further. Cryosections of rat pituitary stored at −20 °C were thawed at RT for 30 min in moist chamber. The tissues were then washed twice with PBS and permeabilized with 0.5% triton X-100 in Tris-Cl (pH- 7.5) for 5 min. GH of the rat pituitary tissues was immunostained with anti-GH antibody (from A. F. Parlow, National Hormone and Pituitary Program, Harbor-ULCA Medical Center, Torrance, CA). Co-staining of tissue amyloid (along with GH) was done using amyloid fibril-specific OC antibody (kind gift of Prof. Charlie Glabe, UC Urvine, USA). Before treating with primary antibody, tissue sections were blocked with 10% NGS + 1% BSA + 0.2% tween 20 in Tris-Cl (pH- 7.5) to prevent non-specific binding. After removing the blocking solution, tissue sections were treated with guinea pig anti-GH antibody (1:1000) and rabbit anti-amyloid fibril OC antibody (1:500), and incubated overnight at 4 °C. Sections were washed thrice with Tris-Cl (pH- 7.5) containing 0.02% tween and the sections were stained with both anti-rabbit Alexa Fluor 488 (Molecular Probes) and anti-guinea pig Alexa Fluor 555 secondary antibody (Dilutions; 1:1000) for 1 h at 37 °C followed by 1 h at RT. Further, the samples were washed with Tris-Cl thrice to remove antibody and were mounted and observed under fluorescence microscope (Leica, DMi8).

### Aggregation of GH in different solution conditions

14 mg of lyophilized GH was dissolved in 5% D-Mannitol with 0.01% azide, pH 5.5 and concentration was adjusted to 500 μM by measuring the absorbance at 280 nm (Jasco model V-650). Several *in vitro* conditions were used for screening the condition required for GH amyloid formation. For studying aggregation in the presence of TFE, TFE was added to GH solution such that, the protein concentration was maintained as 300 μM and the TFE concentrations were varied from 0 to 40% (v/v). To study the effect of ionic strength on GH aggregation, GH solution was treated with various concentrations of NaCl (0.25 M to 3 M) using a 5 M NaCl stock solution prepared in 5% D-Mannitol with 0.01% azide. To study the effect of GAGs; CSA, CSB, C6 S and heparin were selected. 5 mM stock solution of each GAG was prepared in 5% D-Mannitol with 0.01% azide and 10 μl from the stock solution of each GAG was mixed with 90 μl of GH solution in 5% D-Mannitol with 0.01% azide to obtain a final concentration of 500 μM for GH and GAG. Similarly, for studying the aggregation of GH in presence of various bivalent metal ions, 10 μl of 5 mM stock solution of CuSO_4,_ NiSO_4,_ FeSO_4,_ MgSO_4_ and ZnSO_4_ prepared in 5% D-Mannitol with 0.01% azide and was mixed with 90 μl of GH solution in 5% D-Mannitol with 0.01% azide to obtain a final concentration of 500 μM of GH and metal ion. In another experiment, eppendorf tube containing equimolar mixture of GH and metal ions was incubated in presence of 1 mM EDTA as a control. These tubes containing various mixtures of GH were placed into an Echo Therm model RT11 rotating mixture (Torrey Pines Scientific, USA) with a speed corresponding to 50 r.p.m. inside a 37 °C incubator for 15 days. As a control, 500 μM GH in 5% D-Mannitol, 0.01% sodium azide was also incubated. Secondary structural transition and amyloid formation was monitored by CD spectroscopy and ThT binding, respectively on day 0 and on day 15. Finally, electron microscopy was used to analyze the morphology of the aggregates, which showed highest ThT binding.

### CD spectroscopy

For CD measurements, aliquots of GH mixtures were diluted in 5% D-Mannitol to 200 μl in such a way that GH final concentration was 12.5 μM. Quartz cell of 0.1 cm path length (Hellma, Forest Hills, NY) was used to measure the CD spectra using a JASCO 810 instrument. Spectra were recorded over the wavelength range of 198–260 nm at 25 °C. Three independent experiments were performed with each sample. Raw data were processed with smoothing and subtraction of buffer spectra, according to the manufacturer’s instructions.

### ThT binding

Aliquots of GH solutions were diluted in 5% D-Mannitol into 200 μl in such a way that the final concentration of GH was of 12.5 μM. The solution was mixed with 2 μl of 2 mM ThT prepared in 20 mM Tris HCl, pH 8.0. ThT fluorescence was measured immediately after addition of ThT. The fluorescence experiment was carried out either on Horiba-JY (Fluoromax 4) or Shimadzu RF5301 PC, with excitation at 450 nm and emission in the range of 460–500 nm. The slit width for both excitation and emission was kept at 5 nm. The fluorescence intensity values at 482 nm were plotted. The fluorescence signals of all samples were normalized against that of GH + Zn(II) aggregates incubated for 15 days, which was set as 1. Three independent experiments were performed for each sample.

### Transmission electron microscopy

For TEM studies, the GH samples, which showed maximum ThT binding in each conditions and especially samples that were incubated in secretory granule relevant conditions were diluted in distilled water to obtain a concentration of ~50 μM. The samples were then spotted on a glow-discharged, carbon-coated Formvar grid (Electron Microscopy Sciences, Fort Washington, PA) and incubated for 5 min. The grids were then washed with distilled water and then stained with 1% (w/v) aqueous uranyl formate solution. TEM was performed using an electron microscope (FEI Tecnai G2 12) at 120 kV with nominal magnifications in the range of 26,000–60,000. Images were recorded digitally by using the SIS Megaview III imaging system. Two independent experiments were carried out for each sample.

### Isolation and characterization of GH aggregates formed in presence of Zn(II)

For confirming the secondary structure of GH aggregates formed in presence of Zn(II), FTIR spectroscopy was performed. To do so, 500 μl of freshly prepared GH solution (400 μM concentration) was incubated in presence of equimolar concentration of Zn(II) for 15 days. After incubation, 100 μl aliquot of this solution was centrifuged at 90,000 rpm for 1 hr. The pellet containing aggregates were redissolved in 100 μl 5% D-Mannitol, pH 5.5. The concentration of supernatant fraction was calculated by measuring the UV absorbance at 280 nm. The supernatant and pellet fraction was further used for FTIR study. To do so, 5 μl aliquots of each sample (pellet and supernatant fraction) was spotted and dried on translucent pellets of KBr that were made by compressing the grinded KBr powder at the pressure of ~7 ton using a hydraulic pressure pump. For background spectrum, 5 μl of 5% D-Mannitol solution was spotted on another KBr pellet. The pellet was first dried under IR lamp and then kept in a transmission holder and the IR spectra in the range of 1800–1500 cm^−1^ were acquired by using BrukerVertex-80 instrument equipped with DTGS detector. For each spectrum, 32 scans at the resolution of 4 cm^−1^ were recorded. Raw data corresponding to amide-I region (1700–1600 cm^−1^) were deconvoluted by Fourier self deconvolution (FSD) method. The deconvoluted spectra were then subjected to Lorenzian curve fitting procedure by using opus-65 software. Three independent experiments were performed for each sample. Further the supernatant and pellet were also subjected to TEM, ThT and CR binding analysis. The ThT fluorescence signals of all samples were normalized against that of pellet fraction, which was set to 1.

### CR binding

A 5 μl aliquot of 500 μM of various GH aggregates was mixed individually with 80 μl of 5 mM potassium phosphate buffer containing 10% ethanol. Then 15 μl of 100 μM CR solution (filtered through 0.2 μM filter) in 5 mM potassium phosphate buffer containing 10% ethanol was added. After mixing and incubating for 20 mins in dark, absorption spectra was measured in the range of 300–700 nm using JASCO V-650 spectrophotometer. The absorbance values at 540 nm were plotted. For the measurement of the CR-only spectrum, 15 μl of CR solution was mixed with 85 μl of 5 mM potassium phosphate buffer containing 10% ethanol and CR absorbance was recorded. Similar preparations were also used for measuring CR fluorescence. The fluorescence experiment was carried out on Horiba-JY (Fluoromax 4) spectrofluorometer with excitation at 550 nm and emission in the range of 570–650 nm. The slit width for both excitation and emission was kept at 5 nm. Three independent experiments were performed for each sample.

### CR birefringence

The CR birefringence analysis was performed using the diagnostic amyloidal stain kit HT60 from Sigma. GH aggregates were isolated by ultracentrifugation at 90,000 r.p.m for 1 hr and washed once by distilled water. Then, the fibrils were placed in 100 μl of alkaline sodium chloride solution for 20 mins with continuous vortexing for uniform mixing of all fibril particles in solution. The mixture was then centrifuged and pellet fractions were utilized to stain with alkaline CR solution for 20 min with continuous vortexing. The mixtures were centrifuged briefly at 90,000 r.p.m for 1 hr and pellets were washed twice with 500 μl of 20% ethanol. The pellet fractions were resuspended in PBS and then spread evenly onto glass slides and air-dried at room temperature. The slides were analyzed using a microscope (Olympus SZ61 stereo zoom) equipped with two polarizers and a CCD camera.

### X-ray Diffraction of GH fibril formed in presence of Zn(II)

For X-ray diffraction studies, GH fibrils were isolated as mentioned in the previous section and were loaded into a clean pre-dried 0.7 mm capillary tube. Then the sample in glass capillary tube was allowed to dry for 1–2 days under vacuum. The entire capillary with dried protein film was mounted in the path of X-ray beam. The dried sample was placed in x-ray beam at 1.2 kW for 5 min exposure. The images were obtained using Rigaku R Axis IV++ detector (Rigaku, Japan) mounted on a rotating anode. The sample to detector distance was kept 300 mm and the image files were analyzed using Adxv software.

### Zn(II) detection staining of rat pituitary tissues

For detecting chelatable Zn(II) ions, in the anterior pituitary of rat pituitary tissue, FluoZin^TM^ 3-AM (Molecular probes) staining was used. The tissues were initially thawed, washed and permeabilized as mentioned in the previous section for immunohistochemistry. For Zn(II) staining, instead of using PBS, 10 mM HEPES buffer pH 7.4 was used. After blocking the tissue with 5% BSA, the sections were treated by rabbit anti-GH antibody (from A. F. Parlow, National Hormone and Pituitary Program, Harbor-ULCA Medical Center, Torrance, CA) in 1:1000 dilutions and incubated overnight at 4 °C. The excess primary antibody was removed by washing with HEPES buffer and was treated with Alexa 555 anti-rabbit secondary antibody for 2 hr at RT. These tissue sections were then washed with HEPES buffer and incubated with 1 μM FluoZin^TM^ 3-AM (stock prepared in 50% DMSO-20% pluronic and diluted in HEPES buffer) for 1 hr at RT. Further, the sections were washed with HEPES buffer and the tissue was kept in the same buffer for 30 min for hydrolysis of the AM ester for developing the fluorescence. Sections were again washed with HEPES buffer and the stained tissue was mounted and viewed under IX 81 combined with FV-500, Olympus confocal laser scanning microscope.

### NMR and MALDI TOF study of GH

All NMR spectra were acquired with a Bruker Avance 800 MHz spectrometer using a cryogenically cooled triple-resonance probe equipped with z-axis gradient coils. Data were acquired and processed using Topspin 2.1 version and analyzed with Sparky 3.114. DSS (4,4-dimethyl-4-silapentane-1-sulfonic acid) was used as an internal reference for the calibration of proton chemical shifts, whereas nitrogen chemical shift was calibrated indirectly. To perform two-dimensional ^1^H−^15^N correlation HSQC experiments, ^15^N labelled GH was expressed and purified in M9 media, the purification protocol was used as mentioned in the earlier section. 25 mg/ml of lyophilized protein was dissolved in 20 mM phosphate buffer, pH 6.8 and the concentration of the protein was adjusted to 1 mM based on the absorption value at 280 nm. HSQC was performed at 25 °C with ^15^N labeled protein samples prepared such a way that solution contain (90:10) H_2_O/D_2_O ratio. Various sets of ^1^H−^15^N HSQC correlation spectra were recorded with 1 mM GH monomer and GH monomer in presence of equimolar Zn(II). The sample used for NMR study was further taken to perform MALDI TOF (Autoflex speed MALDI TOF, Bruker) analysis. To do so, 2 μl of sample was spotted with synapinic acid (matrix) and analysis was done in linear mode. Two independent experiments were performed for each sample.

### ANS binding study of GH oligomers

For the ANS fluorescence study, 5 μL aliquots of the 400 μM GH in presence and absence of Zn (II) ions was diluted in 200 μL such that the final peptide concentration was ∼10 μM. The solution was taken into a 1 cm path length quartz cuvette, and 3 μL of 5 mM ANS was added to the cuvette followed by 15 min incubation in dark. The ANS fluorescence spectra were recorded using a spectrofluorimeter (Horiba Jobin Yvon (Fluoromax 4)), with an excitation wavelength of 370 nm and emission wavelength in the range of 400 to 600 nm. The excitation and emission slit widths were kept at 5 nm. Three independent experiments were performed for each sample.

### Time-resolved fluorescence studies

For time resolved fluorescence studies, 14 mg of lyophilized GH was dissolved in 5% D-Mannitol with 0.01% azide and concentration was measured by taking the absorbance at 280 nm (Jasco V-650). To 450 μl aliquot of GH monomer solution, 50 μl of 5 mM stock solution of ZnSO_4_ (prepared in 5% D-Mannitol with 0.01% azide) was mixed to obtain a final concentration 500 μM for both GH and Zn(II) ion. 500 μM of GH monomer solution alone was also incubated as control. The tubes containing GH and GH + Zn(II) were placed into an Echo Therm model RT11 rotating mixture (Torrey Pines Scientific) with a speed corresponding to 50 r.p.m. inside a 37 °C incubator for 15 days for aggregation. The GH samples immediately after preparation (day 0) and after 15 days of incubation were taken for Trp intensity decay and anisotropy decay analysis. To do that, both the GH samples were diluted in 5% D-Mannitol, 0.01% azide to get absorption peak of 0.1 at 280 nm to minimize inner filter effect. Time resolved study were performed on IBH Horiba Jobin Yovin time correlated single photon counting (TCSPC) setup with a 295 nm pulsed LED having full width at half maxima of 704 ps. Emission was monitored at 340 nm with a magic angle polarization condition of 54.7° with respect to vertical excitation. For anisotropy decay, excitation and emission polarization angles were changed accordingly and G-factor was calculated taking the ratio of intensities (area under the decay curve) at vertical and horizontal emission polarization when excited with horizontal polarized light. TCSPC data were fitted in biexponential decay law by means of deconvolution technique through IBH DAS 6.2v software. Two independent experiments were performed for each sample.

### Monomer release assay

100 μl aliquot of 15 days-old GH aggregates (400 μM) in presence of Zn(II) were harvested by ultra centrifugation at 90,000 rpm for 1 hr. The pellet was redissolved in 100 μl of 5% D-Mannitol, 0.01% azide. The concentration of the supernatant was measured using the absorbance at 280 nm (Jasco V-650), which was used to calculate the GH concentration in the pellet. The redissolved pellet of 200 μM concentration in 5% D-Mannitol, pH 5.5 was used to perform the monomer release assay using the experimental setup reported previously^41^. Briefly, the pellet solution was transferred into a modified 1.5 ml Eppendorf tube with a hole in its cap, which was sealed with a 50 kDa MWCO dialysis membrane (Spectra/Por, USA). Further the tube containing aggregates were dialyzed against 2 ml of 10 mM Tris HCl buffer, pH 7.4, 0.01% sodium azide in a 15 ml Falcon tube. The tubes were then incubated at 4 °C to prevent evaporation of solutions. To determine the protein concentration outside the membrane (from the 15 ml falcon tube), an aliquot of 100 μl of solution was taken from the releasing medium (buffer outside the dialysis membrane) at regular time intervals and Trp fluorescence was measured by exciting the solution at 290 nm and emission was recorded in the range of 300–500 nm. The absorbance was also measured for the same solution at 280 nm. 200 μM GH solution incubated alone for 15 days was used as a monomer control. In an identical experimental set up, buffer was kept both inside and outside the membrane and the fluorescence and absorbance was measured. This control is used to delineate whether degradation of the dialysis membrane is interfering with the assay. The fluorescence experiment was carried out using Horiba-JY (Fluoromax 4). The slit width for both excitation and emission was kept at 5 nm. After spectra recording, the released solution was returned and the dialysis system was reassembled again. Two independent experiments were performed for each sample.

### Statistical analysis

The statistical significance was determined by one-way ANOVA followed by Newman-Keuls Multiple Comparison post hoc test; *P < 0.05; **P < 0.01; NS P > 0.05.

## Additional Information

**How to cite this article**: Jacob, R. S. *et al.* Amyloid formation of growth hormone in presence of zinc: Relevance to its storage in secretory granules. *Sci. Rep.* **6**, 23370; doi: 10.1038/srep23370 (2016).

## Supplementary Material

Supplementary Information

## Figures and Tables

**Table 1:** Biophysical charcterization of GH aggregates in various conditions after 15 days of incubation.

Conditions		ThT Binding	CR Binding*	CD	TEM	Amyloid
GH		−	−	Helix	Amorphous	−
TFE	10%	+	−	Helix	Amorphous	−
	20%	−	−	Helix	Amorphous	−
	30%	−	−	Helix	ND**	−
	40%	−	−	Helix	ND	−
NaCl	0.25 M	−	−	Helix	ND	−
	0.5 M	−	−	Helix	ND	−
	1 M	+	−	Helix	ND	−
	2 M	+	−	Helix	Amorphous and some ordered aggregates	−
	3 M	+	−	β−sheet	Amorphous and some ordered aggregates	−
Glycosaminoglycans	CSA	−	−	Helix	Ordered aggregate	−
	CSB	−	−	Helix	Amorphous	−
	C6S	−	−	Helix	Amorphous	−
	Hep	−	−	Helix	Amorphous	−
Metals	Cu	+	−	Helix	Amorphous	−
	Zn	+	+	Helix	Short fibrils	+
	Fe	−	−	Helix	Amorphous	−
	Mg	+	−	Helix	Amorphous	−
	Ni	+	−	Helix	Amorphous	−

^*^CR binding based on UV absorption method.

^**^ND, Not Done.
