# Supplementary material for: Amyloid formation of growth hormone in presence of zinc: Relevance to its storage in secretory granules
Source: Sci Rep. 2016 Mar 23;6:23370. doi: 10.1038/srep23370 (PMC4804206; doi:10.1038/srep23370)

Supplementary Information

Amyloid formation of growth hormone in presence of zinc: Relevance to its storage in secretory granules

Reeba S Jacob[1], Subhadeep Das[1,2], Saikat Ghosh[1], Arunagiri Anoop[1], Narendra Nath Jha[1], Tuhin Khan[3], Praful Singru[4], Ashutosh Kumar[1] and Samir K Maji[1a]

[1]Department of Biosciences and Bioengineering, IIT Bombay Mumbai, India 400 076

[2]IITB Monash Research Academy, IIT Bombay, Powai, Mumbai 400076, India.

[3]Department of Chemistry, IIT Bombay Mumbai, India 400076

[4]School of Biological Sciences, National Institute of Science Education and Research, Bhubaneswar, India

[a]samirmaji@iitb.ac.in

Supplementary Figure Legends

Supplementary Figure S1. Prediction of amyloidogenic region in human GH using Zipper DB algorithm. In Zipper DB, regions with high fibrillation propensity are shown with negative free energy. Segments shown in red, with corresponding sequences below indicates highest amyloidgenic region in GH (Zipper DB Accession ID, PDB:1A22_A).

Supplementary Figure S2. CR absorption spectra of various GH aggregates. CR spectra obtained for GH aggregates in presence of various incubating conditions such as (A) TFE, (B) NaCl, (C) GAG, (D) Metal ions

Supplementary Figure S3. CR fluorescence of GH fibrils formed in presence of Zn(II). CR fluorescence showing higher intensity for pellet fraction compared to supernatant fraction. Error bar represents standard error. Statistical significance, $^*P < 0.05$

Supplementary Figure S4. ANS binding of GH oligomers formed in presence of Zn(II). The ANS spectrum of GH oligomers formed in presence of Zn(II) showing an increase in fluorescence intensity with blue shift of λ_{max} ~ 20 nm, indicating high ANS binding. The GH alone sample showed λ_{max} ~ 500 nm indicating less hydrophobic exposed surface area.

Supplementary Figures

Supplementary Figure S1.

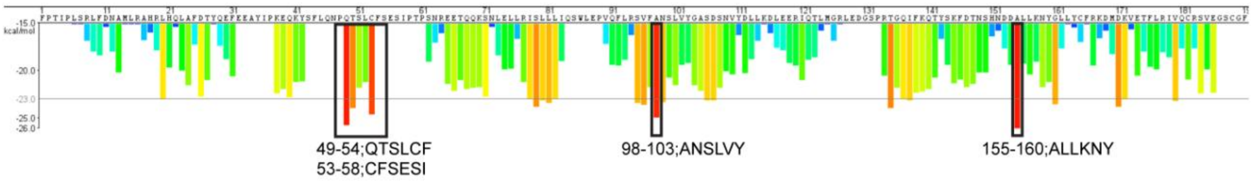

Supplementary Figure S2.

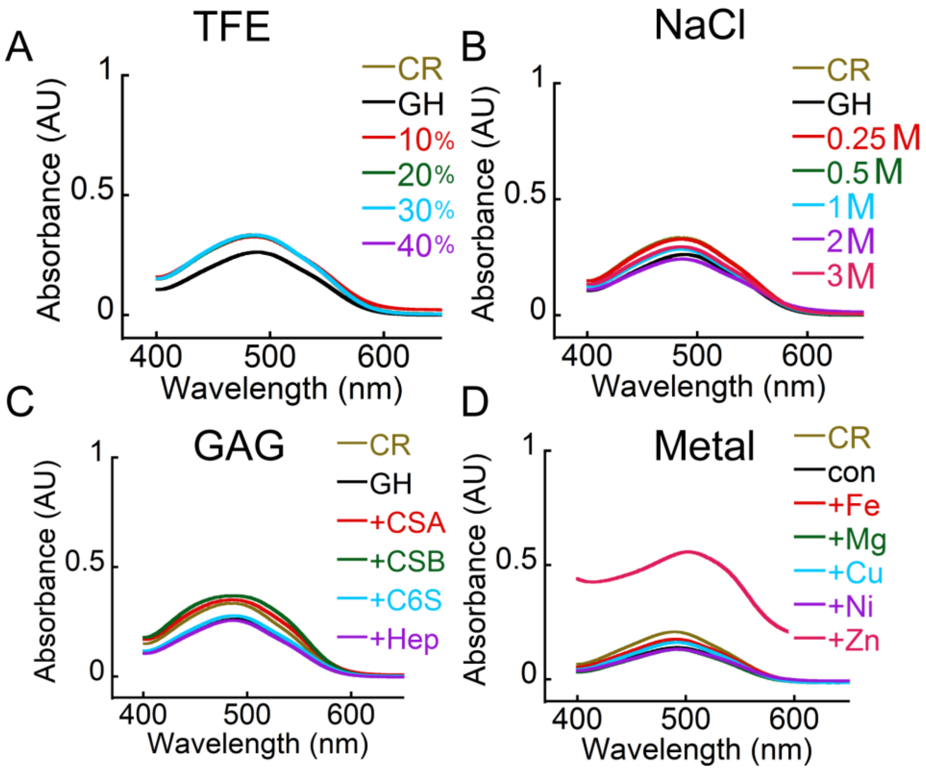

Supplementary Figure S3.

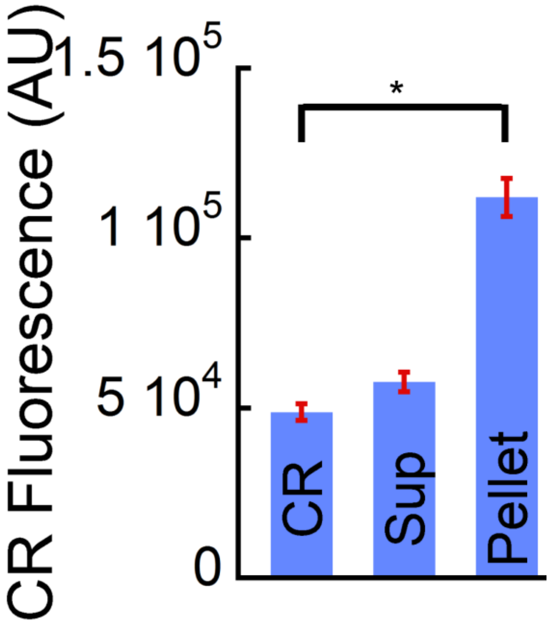

Supplementary Figure S4.

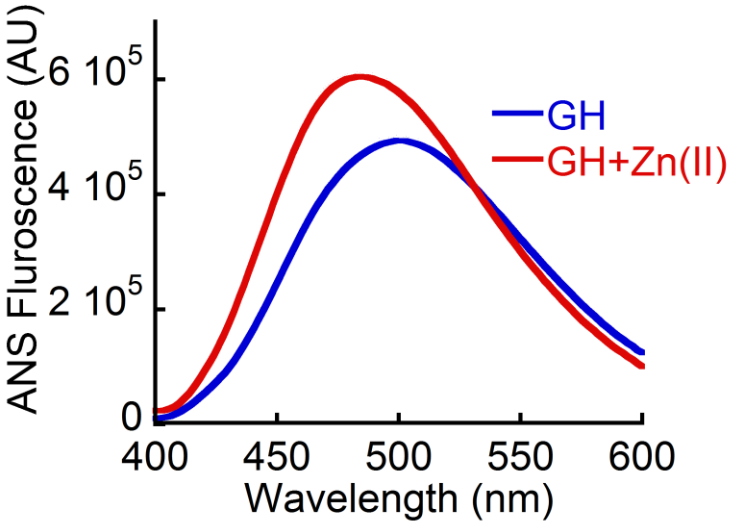

Supplement: Supplementary Information [file srep23370-s1.pdf]
